# Exploring the Impact of Exosomal Cargos on Osteosarcoma Progression: Insights into Therapeutic Potential

**DOI:** 10.3390/ijms25010568

**Published:** 2024-01-01

**Authors:** Claire C. Chen, Claudia A. Benavente

**Affiliations:** 1 Department of Pharmaceutical Sciences, University of California, Irvine, CA 92697, USA; claire.chen@uci.edu; 2 Department of Developmental and Cell Biology, University of California, Irvine, CA 92697, USA; 3 Chao Family Comprehensive Cancer Center, University of California, Irvine, CA 92697, USA

**Keywords:** exosomes, extracellular vesicles, osteosarcoma

## Abstract

Osteosarcoma (OS) is a primary malignant bone tumor with high metastasis. Poor prognosis highlights a clinical need for novel therapeutic strategies. Exosomes, also known as extracellular vesicles, have been identified as essential players in the modulation of cancer. Recent studies have suggested that OS-derived exosomes can drive pro-tumorigenic or anti-tumorigenic phenotypes by transferring specific cargos, including proteins, nucleic acids, and metabolites, to neighboring cells, significantly impacting the regulation of cellular processes. This review discusses the advancement of exosomes and their cargos in OS. We examine how these exosomes contribute to the modulation of cellular phenotypes associated with tumor progression and metastasis. Furthermore, we explore the potential of exosomes as valuable biomarkers for diagnostics and prognostic purposes and their role in shaping innovative therapeutic strategies in OS treatment development.

## 1. Introduction

Osteosarcoma (OS) is the most common malignant bone tumor in children, adolescents, young adults, and adults aged over 60 [1]. It is characterized by the production of osteoid by malignant osteoblasts, often located in the metaphysis of long bones, with the distal femur, proximal tibia, and proximal humerus being the most prevalent locations [2]. The most metastatic site for OS is the lungs, followed by distant bones, with 20% of patients presenting pulmonary metastasis at initial diagnosis, and it is considered to be associated with poor prognosis [3]. Current treatment regimens for OS consist of neoadjuvant and adjuvant chemotherapy, surgical resection, and ongoing research focusing on targeted therapies and immunotherapy [4,5]. The chemotherapy MAP (cisplatin, doxorubicin, and methotrexate) treatment is widely acknowledged as the most established and effective treatment approach for metastatic and nonmetastatic OS [6]. The 5-year survival rate for OS is 60–70% for localized disease and ~20% for recurrent or metastatic OS [7,8]. Despite significant advancement in chemotherapeutic agents leading to improved prognosis of OS, the clinical outcomes for patients with OS remain suboptimal due to the prevalent development of multi-drug resistance. In addition, lung metastasis and multiple relapses are contributing factors and primary causes of death [3]. Therefore, it is crucial to understand the pathogenesis of OS and the underlying mechanisms of chemoresistance to identify potential biomarkers for the development of novel and more effective therapeutic and enhance clinical outcomes.

**Figure 1:**
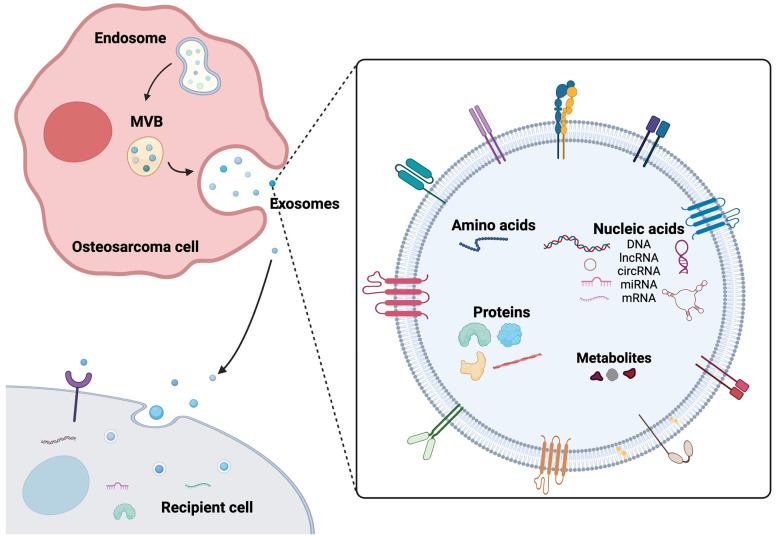
Exosome biogenesis, secretion, and composition. Exosomes originate from endosomes and are released through the fusion of multivesicular bodies (MVBs). The fusion of MVBs with the plasma membrane results in the release of exosomes, which transport a diverse range of cargos, including proteins, DNA, RNAs, and metabolites to recipient cells.

Extracellular vesicles (EVs) are membrane-bound phospholipid bilayer vesicles and are naturally secreted by cells. EVs can be categorized into apoptotic bodies (1000–5000 nm in diameter), microvesicles (150–1000 nm in diameter), and exosomes (30–150 nm in diameter) according to their sizes and intracellular origin [9]. Apoptotic bodies are generated during apoptotic cell death; microvesicles are released by outward budding of the plasma membrane; exosomes are generated by the formation of intracavitary vesicles (ILV) within multivesicular bodies (MVBs), the exocytosis of MVBs, and are released into extracellular space when MVBs fuse with the plasma membrane (Figure 1) [10,11,12]. Exosomes can be found in various body fluids, including blood, urine, saliva, plasma, serum, milk, bile, amniotic fluid, and cerebrospinal fluid [13,14,15,16,17,18,19,20,21]. These vesicles contain lipids, proteins, nucleic acids, metabolites, and the composition of exosomes reflects the molecular content of the cell from which they originate, making them valuable carriers of information about the cell’s status, function, and cellular environment. Exosomes play an important role in cell-to-cell communication, facilitating the transfer of their cargo to neighboring or distant recipient cells. Through this transfer of biomolecules, exosomes have a remarkable ability to mediate diverse cellular processes, such as gene expression, immune regulation, signal transduction, tissue repair and regeneration, epigenetic reprogramming, and cancer progression [22,23,24,25,26,27,28,29,30,31,32].

**Figure 2:**
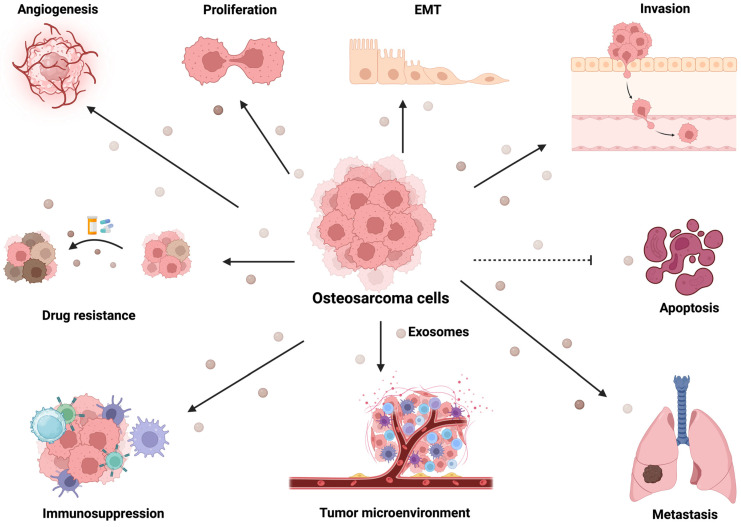
Exosomes derived from OS promote tumor progression. Exosomes secreted from OS regulate cancer progression through various mechanisms. These include the induction of angiogenesis, cell proliferation, EMT, invasion, apoptosis, drug resistance, immunosuppression, modulation of the tumor microenvironment, and facilitation of metastasis. The specific exosomes that mediate these biological functions are listed in Table 1.

In recent years, accumulating evidence highlights the significant role of exosomes in the progression of OS by facilitating intracellular communication. This review explores the role of exosomes in OS, focusing on their involvement in drug resistance and potential diagnostic and prognostic applications. We conducted a literature search up to September 2023 on PubMed and Google Scholar, using the search terms “osteosarcoma, exosomes, extracellular vesicles, drug resistance, biomarkers, and therapeutics.” Included in the review were studies focusing on exosomal cargos and their roles in OS progression, examining the potential applications for biomarkers or therapeutic interventions. Studies that did not align with the scope of this review and non-peer-reviewed articles were excluded. OS-derived exosomes possess a distinctive molecular cargo comprising oncogenes, oncoproteins, and microRNAs (miRNA), which have been implicated in driving metastasis, angiogenesis, and other critical aspects of OS progression [33,34,35,36]. The diverse roles of exosomes in the hallmarks of cancer are summarized in Figure 2 and Table 1. Currently, exosomes are explored as versatile tools, serving as biomarkers for diagnosis and prognosis, potential targets for therapeutics, and vehicles for drug delivery [37,38,39]. The scope of this review encompasses these various aspects, including the transfer of drug resistance through exosomes, the molecular mechanisms involved, and the potential of exosomal biomarkers for OS.

## 2. Osteosarcoma-Derived Exosomes Mediate Angiogenesis

Angiogenesis facilitates OS progression by forming new blood vessels that provide the tumor with essential nutrients and oxygen, thereby fostering its expansion and metastasis. The regulation of angiogenesis in OS involves a delicate equilibrium between pro-angiogenic and anti-angiogenic factors, with the OS often exhibiting overexpression of pro-angiogenic factors [36,84,85]. These pro-angiogenic factors can induce neoplastic vascularization and control vascular permeability to support tumor growth [86,87]. Several studies suggest that OS-derived exosomes support tumor progression, at least in part, through the release of pro-angiogenic factors.

Perut et al. investigated whether extracellular acidity triggered by OS can stimulate exosomes secretion and alter their pro-angiogenic properties. Several pro-angiogeneic factors were identified in the exosomal cargo, including proteins (VEGF, PDGF-AA, CD26, CD105, endostatin, ET-1, PAI-1, THBS1, TIMP-1, PEDF, uPA, ANG-2, TF3, PTX3, and HB-EGF) and miRNAs (miR-10b-5p, miR-106a-5p, miR-125b-5p, miR-143-3p, miR-145-5p, miR-146a-5p, miR-150-5p, miR-21-5p, miR-26a-5p, miR-27a-3p, miR-382-5p, miR-92a-3p, and miR-93-5p) [40]. A study by Raimondi et al. showed that OS cell-derived exosomes induce endothelial cells to release pro-angiogenic factors such as VEGF-A, IL-6, and IL-8, thereby promoting endothelial cell tube formation and modulating OS progression [33]. In addition, exosomal miR-21-5p and miR-148a-3p have been identified to modify the behavior of osteoclast and endothelial cells. These miRNAs regulate osteoclastogenesis and the secretion of pro-angiogenic factors, stimulating the process of angiogenesis [33]. Other miRNAs and long noncoding RNAs from OS exosomes are involved in angiogenesis. For example, miR-25-3p is highly expressed in OS-derived exosomes and actively facilitates capillary formation and the invasion of vascular endothelial cells [56]. OS-derived exosomes carrying long noncoding RNA (lncRNA 227, also known as EWSAT-1) have been discovered to promote angiogenesis by enhancing the sensitivity/reactivity of vascular endothelial cells and by increasing the secretion of angiogenic factors [72]. The abnormal upregulation of lncRNA OIP5-AS1, secreted by OS cells, exerts regulatory effects on autophagy through miR-153 and ATG5, consequently leading to enhanced angiogenesis [73]. Additionally, OS-derived exosomal miR-199-5p has been found to exert a tumor-suppressive role in OS, where overexpression of miR-199-5p modulates VEGFA and subsequently inhibits proliferation, migration, and neovascularization of human endothelial cells [57].

These studies and the identified proteins and miRNAs within the exosomal cargo point at a complex network of molecules influencing neoplastic vascularization and vascular permeability. The modulation of endothelial cells by OS cell-derived exosomes, including the induction of pro-angiogenic factors and the regulation of miRNAs, further emphasizes their impact on angiogenesis. Overall, these studies highlight the diversity of regulatory mechanisms that contribute to angiogenesis in osteosarcoma and suggest that targeting these exosomal components could be a potential therapeutic strategy for inhibiting angiogenesis and, consequently, impeding osteosarcoma progression.

## 3. Exosomal Cargos Mediate Metastasis of Osteosarcoma

Substantial in vitro and in vivo evidence strongly supports the important role and potential clinical relevance of exosomal cargo in both the development and progression of OS. In a recent study, Jerez et al. investigated exosomal cargos from human OS (SAOS2, MG63, U2OS, HOS, and 143B) or osteoblast cell lines (hFOB 1.19 cells) with different metastatic potential. Using miRNA sequencing profiling, the researchers identified four miRNAs (miR-21-5p, miR-143-3p, miR-148a-3p, and 181a-5p) that were significantly enriched, with higher expression levels in metastatic SAOS2 cells compared to those of nonmetastatic MG63 cells. Gene ontology analysis suggests these miRNAs may regulate cell adhesion and apoptosis [58]. However, no phenotypic validation was included in this study. Support for some of these observations comes from other independent studies and are contradicted by others. Indeed, miR-21-5p is considered an oncomir in solid and hematological malignancies [88,89,90], where exosomal miR-21-5p mediates crosstalk within the tumor microenvironment (TME) to prepare the metastatic niche. Inhibition of miR-21-5p leads to decreased proliferation and metastasis of OS by targeting PTEN and modulating the TGF-β1 signaling pathway [33,58,91,92,93]. OS-derived exosomal miR-1307 regulates AGAP1 and induces OS tumorigenesis [59]. In vitro and in vivo studies have demonstrated that exosomes secreted by OS, carrying miR-195-3p, enhance the proliferation and invasion of 143B cells [60]. Gong et al. demonstrated that miR-675 is significantly upregulated in exosomes derived from metastatic OS cells in comparison to nonmetastatic OS cells, leading to enhanced migration and invasion of osteoblasts through the downregulation of calneuron 1 (CALN1) expression [61]. Exosomes released by cancer-associated fibroblasts transfer miR-1228 to OS cells, leading to the promotion of OS migration and invasion through the inhibition of endogenous suppressor of cancer cell invasion (SCAI) expression [62]. The transfer of exosomal miR-208a obtained from bone marrow mesenchymal stem cells (BMSCs) to OS cells was observed to promote OS cell proliferation, migration, and clonogenicity while inhibiting apoptosis. This effect was attributed to the downregulation of programmed cell death protein 4 (PDCD4) and activation of the Hippo and ERK1/2 signaling pathways [63]. Likewise, exosomal miR-769-5p was identified to promote OS cell proliferation and metastasis in vitro and in vivo by downregulating Dual-specific phosphatase 16 (DUSP16) and activating the JNK/p38 MAPK (mitogen-activated protein kinase) signaling pathway [64]. Upregulation of oncogenic miR-25-3p from OS-derived exosomes enhances invasion and proliferation of umbilical vein endothelial cells by targeting Dickkopf WNT signaling pathway inhibitor 3 (DKK3) [56]. Interestingly, studies on exosomal miR-143 on OS progression show contradictive results. While next-generation sequencing analysis revealed that exosomal miR-143 from OS cells is expressed at higher levels in metastatic SAOS2 cells [58], other studies suggest miR-143 could suppress tumor metastatic potential by targeting mitogen-activated protein kinase 7 (MAPK7) [94,95]. A study by Li et al. reported that up-regulation of miR-143 could dampen OS cell migration, proliferation, and invasive capability and induce apoptosis via caspase3 activation by targeting Bcl-2 [96]. Osaki et al. observed that administration of miR-143 suppresses spontaneous lung metastasis of OS cells in mice by targeting MMP-13 [97]. These apparently contradictive results can be easily reconciled considering that SAOS2 cells are low-metastatic cells [98].

In addition to oncogenic miRNAs present in exosomes derived from OS cells, some studies have identified several exosomal miRNAs that exhibit tumor-suppressive effects. For example, Zhang et al.’s study highlighted that exosomes derived from adipose tissue-derived mesenchymal stromal cells (AD-MSCs) are abundant in miR-101, which can be taken up by OS cells. By directly down-regulating B-cell lymphoma 6 (BCL6), miR-101 acts as a tumor suppressor, effectively inhibiting metastasis in vivo with minimal observed side effects [65]. Similarly, exosomes containing miR-206 from bone marrow MSCs could suppress OS tumor progression and induce their apoptosis by targeting transformer 2β (TRA2B) [66]. Exosomal miR-1913 derived from bone marrow MSCs (BMSCs) can shuttle to OS cells, exerting a suppressive effect on cell proliferation, invasion, and migration by negatively targeting Neurensin-2 (NRSN2) [67]. Similarly, BMSCs-derived exosomes carrying miR-150 was found to suppress OS cell proliferation, migration, and invasion, and induce apoptosis by targeting IGF2BP1 [68]. Furthermore, exosomal miR-15a obtained from serum was suggested to inhibit arrest cell cycle progression and OS growth in vitro through suppression of GATA2/MDM2 signaling via the p53q signaling [69]. By negatively regulating ZEB1, exosomal miR-144-3p could mediate ferroptosis, suppressing OS metastatic phenotypes [70].

Besides exosomal miRNAs mediating OS metastatic process, non-coding RNAs (ncRNAs), such as long non-coding RNAs (lncRNAs), are also involved in OS progression. For instance, Zhao et al. uncovered that the lncRNA plasmacytoma variant translocation 1 (PVT1), originating from bone marrow MSCs, can be transferred to OS cells, facilitating OS metastasis and tumor growth through miR-183-5p sponging and inhibition of ERG ubiquitination [74]. The lncRNA LINC00852 is highly expressed in AXL receptor tyrosine kinase (AXL)-associated exosomes and plays a role in upregulating AXL expression by competitively binding with miR-7-5p, leading to increased proliferation, migration, and invasion of OS cells [75]. Zhang et al. sought to elucidate the impact of macrophage-derived exosomal lncRNA in OS development. They identified exosomal lncRNA LIFR-AS1, released from macrophage exosomes to OS cells, enhances OS proliferation, invasion, and apoptosis suppression by sequestering the miR-29a/NFIA pathway [76]. Another study reported that lncRNA cancer susceptibility 15 (CASC15) is upregulated in OS plasma exosomes, and knockdown of CASC15 has been found to impede tumor growth both in vitro and in vivo by targeting the miR-338-3p/RAB14 axis [77]. The exosomal lncRNA MALAT1 derived from BM-MSC serves as an additional target, facilitating OS migration, invasion, and proliferation via the MALAT1/miR-143/NRSN2/Wnt/β-catenin axis [78]. More recently, exosomal lncRNA X inactive specific transcripts (XIST) bone marrow MSCs promotes OS progression and lung metastasis by down-regulating miR-655 while upregulating ACLY. This leads to heightened lipid synthesis and the activation of the β-catenin signaling pathway, ultimately nurturing malignant OS phenotypes [79]. Moreover, Li et al.’s study discovered the trafficking of exosomal circRNA within TME, showing that exosomal circ_0000190 can be transported from normal cells to OS cells, leading to the alleviation of the malignant phenotypes of OS through the induction of miR-767-5p, which in turn modulate TET1, effectively hindering the progression of OS [82]. Though these studies demonstrated evidence of exosomal cargos in subcutaneous xenograft model, further investigation is warranted to assess the impact of exosomal cargos in an orthotopic xenograft OS model for clinical relevance.

Extensive evidence underscores the significant contribution of exosomal proteins to the development and progression of OS. For instance, Baglio et al. demonstrated that exosomes derived from highly metastatic OS cells display a substantially higher level of membrane-associated form of TGF-β. These exosomes can be internalized by MSCs, inducing the production of proinflammatory cytokine IL-6, and resulting in alternations of pro-metastatic and pro-tumorigenic phenotypes in vivo [41]. An exosomal protein cargo collagen type VI alpha (COL6A1), originating from OS cells, exhibits the capability to transfer from OS cells to cancer-associated fibroblasts (CAFs). This transfer triggers the activation of CAFs, prompting them to release IL-6 and IL-8. The activated CAFs play a crucial role in driving OS metastasis through the secretion of TGF-β [42]. A study by Zhong et al. revealed that the exosomal Rab22a-NeoF1 fusion protein, in conjunction with its binding partner PYK2 from OS cells, could induce RhoA activation to support the pre-metastatic niche formation through the recruitment of bone marrow-derived macrophages. This process also mediates the polarization of M2 macrophages, subsequently promoting lung metastasis [43]. Exosomes derived from bone marrow MSCs containing lymphocyte cytosolic protein 1 (LCP1) have been shown to foster OS proliferation, epithelial-mesenchymal transition (EMT) process, and metastasis through neuregulin receptor degradation protein-1 (Nrdp1) degradation and activation of the JAK2/STAT3 pathway, while its expression is negatively regulated by a tumor-suppressing miRNA miR-135a-5p [44]. Exosomes derived from adipose MSCs were found to enhance migration, proliferation, and invasion of OS in vitro and in vivo by delivering COLGALT2 [45]. Silencing exosomal cargo of hydrogen peroxide inducible clone 5 (Hic-5) from OS cells could inactivate the Wnt/β-catenin signaling pathway, consequently restraining cell proliferation and triggering apoptosis of OS cells [46]. Likewise, suppressing ATG5 in OS cells could mitigate tumor progression and the metastatic potential associated with exosomes MSCs derived exosomes [47]. A study by Macklin et al. proves exosomes derived from highly metastatic clonal variants of the KHOS cell line carry unique protein cargos, such as NPM1, CCT2, CCT4, CCT6A, CCT8, VIM, CLTC, COL6A2, HNRNPC, PKM, ACTN4, MYH10, PAICS, VCP, ANXA1, and ACLY. These cargos induce migratory and invasive phenotypes in poorly metastatic counterparts while preferentially colonizing the lungs, thus driving metastatic behavior within the same cell line [48]. Using a multi-omics approach, Endo-Munoz et al. highlighted that the urokinase plasminogen activator (uPA) and the uPA receptor (uPAR) are highly elevated in OS-derived exosomes, where autocrine and paracrine uPA/uPAR axis activation plays a role in transitioning from a nonmetastatic to a metastatic phenotype [49]. More recently, we discovered a novel target Ubiquitin-like, containing PHD and RING finger domains, 1 (UHRF1), which participates in exosome release and governs uPA production, thereby regulating OS migratory process [99]. Additionally, an increased expression of exosomal cargos, such as programmed death-ligand 1 (PD-L1) and N-cadherin, was identified in exosomes originating from OS cells, enhancing pulmonary metastasis in metastatic mouse models [50].

Collectively, these findings highlight exosomal cargos in signaling pathways, providing insight into their pivotal role in driving OS progression and metastasis. However, while a plethora of associations between exosomal cargo and OS pro-metastatic phenotypes have been reported, a critical gap exists in terms of functional studies that mechanistically validate these associations. Understanding the cause–effect relationship is crucial for establishing the clinical relevance of these findings. Further integration of functional studies and addressing OS heterogeneity will contribute to the development of more targeted and effective therapeutic strategies.

## 4. Osteosarcoma-Derived Exosomal Cargos Mediated Immune Regulation

The interplay between tumor and immune cells within the TME contributes to OS progression and metastasis. The immune microenvironment highlights the complex crosstalk network influencing disease outcomes and therapeutic responses. Exosomes from OS cells can release growth factors, cytokines, and chemokines to facilitate intracellular communication and reprogram recipient immune cells [33]. Previous studies reveal a distinct difference in exosomal cargos altering the immune microenvironment in OS. For example, Troyer and colleagues reported exosomes carrying immunosuppressive protein cargos such as TGF-β, α fetoprotein, and heat shock proteins in OS cells compared to exosomes derived from healthy osteoblasts. They also observed that the activation and proliferation of CD4^+^ and CD8^+^ T cells were attenuated, while the expression of regulatory (FOXP3^+^) CD4^+^ was elevated, resulting in immune invasion [51]. Among the array of immune cells, tumor-associated macrophages (TAMs)—the predominant immune cells infiltrating OS—are increasingly recognized as pivotal regulators of inflammation, neoangiogenic process, and evasion of immune surveillance [100,101,102]. The dysregulation in the M1-M2 polarization of macrophages, with M1 exhibiting an anti-tumor phenotype and M2 promoting tumor-related phenotypes, governs the progression of cancer pathogenesis [103]. A recent study by Wolf-Dennen et al. demonstrated that exosomes secreted from metastatic OS cells induce macrophage M2 polarization by upregulating the expression of M2-associated cytokines and chemokines, including IL-10, TGFβ2, and CCL2. This process leads to an establishment of immunosuppressive TME and regulates phagocytosis and macrophage-mediated tumor killing [34].

Moreover, it is observed that exosomes derived from OS cells transport cargo T cell immunoglobulin and mucin domain 3 (TIM-3) to promote TAM differentiation to M2 phenotype, which facilitates OS migration, invasion, EMT and distinct metastasis in vitro and in vivo [52]. Similarly, the exosomal lncRNA ELFN1-AS1 derived from OS cells has been shown to transfer to macrophages, where it modulates M2 polarization through sponging miR-138-5p and miR-1291 and subsequently promoting tumor growth [80]. An additional exosomal cargo, Rab22a-NeoF1 fusion protein, has been reported to regulate the activation of signal transducer and activator of transcription 3 (STAT3), leading to M2 macrophage polarization and facilitation of pulmonary metastasis [43]. Likewise, Liu and colleagues demonstrated that the connection between OS malignant phenotypes and M2 polarization of TAMs is controlled by exosomal miR-221-3p, where miR-221-3p released from M2-polarized TAMs exosomes could augment tumor growth while reducing apoptosis through SOCS3/JAK2/STAT3 signaling [71].

Given that exosomes mediate crosstalk within the TME, the role of exosomes secreted by OS in immune regulation has been explored. Strikingly, recent evidence has highlighted cancer cell-derived exosomes carrying tumor-associated antigens and immunosuppressive cargos, such as FasL, PD-L1, and TGFβ, in facilitating tumor immune evasion [35,51,104]. In particular, Zhang et al. investigated the role of exosomal cargo PD-L1 in immune surveillance. Their study revealed that OS-derived exosomes carrying PD-L1 cargo could dampen T-cell activity and mediate tumor growth in vitro and in vivo [53]. These exosomal cargos have an indirect impact on promoting immune escape.

Altogether, there is a clear interplay between exosomal cargo and the immune microenvironment in OS. Modulating the release of exosomal cargo holds promise as an innovative approach to mitigate the immunosuppressive TME and improve the effectiveness of immunotherapy for OS.

## 5. Exosomal Cargos Mediate Osteosarcoma Drug Resistance

Neoadjuvant chemotherapy combined with surgery is the current standard of care for OS. Although chemotherapy helps reduce the tumor burden in OS patients, developing multi-drug resistance (MDR) presents a significant challenge in treating OS, resulting in metastasis and unfavorable prognosis. Previous studies have suggested exosomes mediate MDR in various types of cancer, such as glioblastoma, non-small cell lung cancer, ovarian cancer, gastric cancer, colorectal cancer, and breast cancer [105,106,107,108,109,110]. In OS, multiple studies have demonstrated that MDR arises from exosomes released from drug-resistant parental cells [54,81,83]. Torreggiani et al. demonstrated that exosomes derived from doxorubicin-resistant OS cells can be internalized by recipient cells, shuttling multi-drug resistance-associated protein 1 (MDR-1) mRNA and drug efflux pump P-glycoprotein (P-gp) to sensitive cells, thus propagating doxorubicin-resistant traits [54]. Similarly, the expression of exosomal circ_103801, originating from cisplatin (CDDP)-resistant MG63 cells, was elevated compared to the expression in MG63 cells. This transfer of circ_103801 from CDDP-resistant cells to chemosensitive OS cells. The transfer of circ_103801 from CDDP-resistant cells to chemo-sensitive OS cells was found to suppress apoptosis, reduce drug sensitivity to CDDP, increase the levels of multi-drug resistance-associated protein 1 and P-gp, and significantly contribute to the development of chemoresistance [83]. The exosomal lncRNA ANCR from doxorubicin-resistant KHOS/U2OS cells has been shown to regulate drug sensitivity to doxorubicin, as evidenced by the inability of exosomes with lncRNA ANCR knockdown to induce doxorubicin chemoresistance in mice [81]. Exosomal CCCTC-binding factor (CTCF) from CDDP-resistant OS cells activates the autophagy signaling pathway through the IGF2-AS/miR-579-3p/MSH6 axis, resulting in enhanced CDDP resistance and promoting tumor formation [55]. Given the established role of the PD-1/PD-L1 axis in promoting chemoresistance in various cancers including breast cancer, B-cell lymphoma, and small cell lung cancer, a study by Yati et al. uncovered that OS cells treated with doxorubicin can prompt the release of exosomes that mediate PD-L1 expression in OS cells through IL-1 signaling pathway [111,112,113,114]. The study suggested a relationship between chemoresistant OS-derived EVs and proinflammatory cytokine regulation. However, additional investigations, particularly those focusing on phenotypic analysis, are imperative to decipher the mechanism by which EVs regulate PD-1/PD-L1. Collectively, these findings emphasize the functional role of exosomes from chemoresistance OS cells in mediating drug resistance through mechanisms such as drug efflux, exosomal cargo delivery, and RNA transport, suggesting that targeting specific exosomal cargos could potentially help overcome MDR in OS.

## 6. Exosomes as Biomarkers in Osteosarcoma

**Figure 3:**
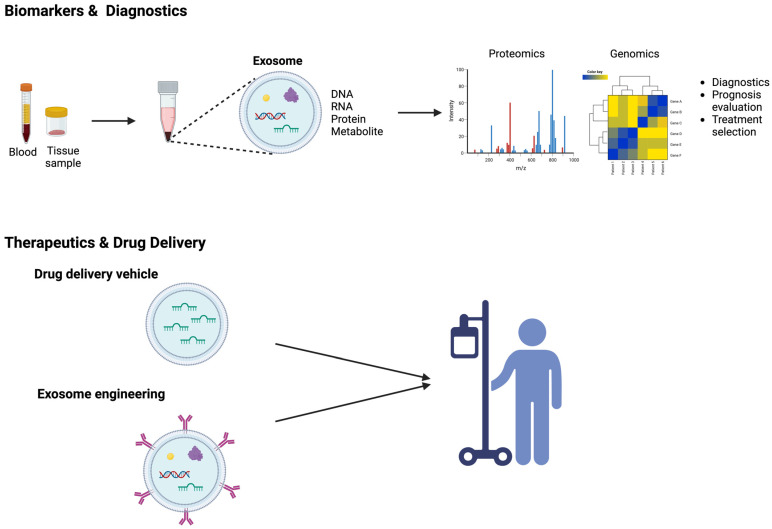
Exosomes as emerging biomarkers and therapeutic tools in OS. Exosomes can be extracted from body fluids or tissues, and exosomal cargos including DNAs, RNAs, proteins, and metabolites can be analyzed through proteomics or genomics to evaluate OS diagnosis, prognosis, and response to treatment. Additionally, exosomes serve as versatile therapeutic tools, functioning as drug delivery vehicles or facilitating personalized medicine through exosome engineering.

Exosomes play a crucial role as carriers, shuttling various cargo molecules, including proteins, nucleic acids, and signaling molecules, contributing to the intricate process of tumorigenesis and cancer progression. To date, there is no robust biomarker available for predicting prognosis and metastasis in OS. Given the unique and complex composition of exosomes, along with their widespread presence in various tissues and biological fluids, cancer exosomes have been explored extensively as a valuable tool for biomarker applications in diagnostics, prognostic assessments, and tumor monitoring. Indeed, exosomes exhibit distinct molecular profiles between normal and pathological states, and numerous studies have discovered that exosome-derived cargos serve as potential biomarkers for OS. Exosomes derived from the blood or tissues of OS patients can be isolated, purified, and characterized by analyzing their cargos through genomics or proteomics analysis (Figure 3 and Table 2). The molecular signature associated with OS pathogenesis can be identified by scrutinizing the RNA or protein compositions presented in the exosomal cargo. Exosomal miRNAs have been predominantly exploited as biomarkers for the diagnosis of cancer. For example, Ye and colleagues investigated miRNA profiling in exosomes derived from plasma samples of OS patients and healthy individuals, revealing differential expression of 57 miRNAs, with 20 being upregulated and 37 downregulated. Notably, miR-92a-3p, miR-130a-3p, miR-195-3p, miR-335-5p, and let-7i-3p were significantly higher in OS patient exosomes compared to those from healthy controls [60]. In a clinical trial (NCT03108677), using plasma-derived exosomes as a liquid biopsy approach revealed significant transcriptomic alterations, including a higher tumor mutation burden, gene fusions, and aberrant gene expression in metastatic OS patients compared to primary OS. This suggests that the transcriptomic profiling of plasma exosomes holds the potential to differentiate the metastatic potential in OS patients, offering a novel approach for monitoring metastasis [115]. Gong et al. found a significantly higher expression of exosomal miR-675 in metastatic OS patients’ serums associated with prognosis [61]. Similarly, the miR-25-3p level was remarkably elevated in both the serum and tissues of OS patients and is inversely correlated with the clinical prognosis [56,116]. A separate study reported a significant decrease in the expression of plasma exosomal miR-101 in OS patients compared to healthy individuals. Notably, this decrease was even more pronounced in metastatic OS patients than in those without metastasis. This observation underscores the possibility of utilizing circulating exosomal miR-101 as a potential diagnostic biomarker for detecting OS metastasis [65]. In addition, a study by Xu et al. reported serum exosomal mRNA and miRNA profiles unveiling distinct expression patterns between OS patients exhibiting favorable and unfavorable responses to chemotherapy. Specifically, they discovered upregulation of mRNA expression in Annexin2, CDC5L, Smad2, and P27, while CIP4, MTAP, PEDF, and WWOX were found to be downregulated in OS patients with poor chemotherapeutic responses. Further, the expression levels of exosomal miR-135b, miR-148a, miR-27a, and miR-9 were elevated, whereas miR-124, miR-133a, miR-199a-3p, and miR-385 were observed to be down-regulated in OS patients with inadequate chemotherapeutic response, indicating exosomal RNA or miRNA can be used for potential biomarkers in categorizing different levels of chemotherapy sensitivity in OS [117]. Using the NGS approach, Cuscino et al. identified eight putative miRNA sequences in the exosomes derived from OS cell lines. Among these, five miRNA candidates exhibited differential expression in liquid biopsy samples from a small OS patient cohort compared to controls. These miRNA targets might represent potential biomarkers, yet further research is imperative to investigate the underlying pathological mechanisms associated with these miRNA candidates [118]. Serum-derived exosomal circ_103801 has been found to be upregulated in OS patients, and survival analysis revealed that higher circ_103801 is associated with shorter overall survival, suggesting a potential role for circ_103801 as a prognostic biomarker for OS [83]. Other exosomal non-coding RNA targets, circ_0056285, circ_0000190, and lncRNA CASC15 have shown significantly increased expression in the serum or plasma of OS patients, displaying strong diagnostic potential [77,82,119].

Research has also suggested that exosomal protein cargos can serve as promising biomarkers for predicting OS prognosis. In their study involving 146 patients with OS, Wang et al. identified a significant correlation between the expression of plasma exosomal sentrin SUMO-specific protease 1 (SENP1) and factors such as tumor size, tumor location, necrosis rate, pulmonary metastasis, and surgical stage. Additionally, patients with higher levels of SENP1 expression experienced less favorable outcomes in both overall survival and disease-free survival (DFS) than patients with lower SENP1 expression [120]. Using surface-enhanced Raman scattering (SERS) and matrix-assisted laser desorption/ionization time-of-flight mass spectrometry (MALDI-TOF MS) allows researchers discerning of distinct plasma exosome profiles between OS patients and healthy individuals. Additionally, the MALDI-TOF MS analysis of plasma exosomes from OS patients further identified that seven exosomal protein cargos (IGLV2-23, IGLV4-3, IGLV1-51, IGKV3-15, IGHV4-4, IGLV4-60, and HBA1) are associated with lung metastasis [121,123]. It was observed that the expression of serum exosomal TGFβ was upregulated in OS patients compared to health controls [41]. Likewise, another study demonstrated that the expression level of plasma exosomal PD-L1 and N-cadherin is significantly upregulated in OS patients with lung metastasis, in contrast to those without metastasis. This suggests the possibility of these biomarkers as potential candidates for predicting pulmonary metastasis and tumor progression in OS [50].

In addition to utilizing exosomal RNA and protein cargos as biomarker tools, recent research has begun to explore the potential of exosomal DNA cargos as valuable cancer biomarkers. Thakur and colleagues demonstrated that double-stranded DNA in exosomes derived from tumor cells carries the mutational status of the parent cells, highlighting its potential as a surrogate for detecting genetic mutations in cancer patients [124]. A recent study by Cambier et al. revealed elevated expression of repetitive element DNA, as opposed to RNA levels, within serum exosomes from OS patients compared to the serum exosomes from control subjects. This included the upregulation of HSATI, HSATII, LINE1-P1, and Charlie 3 [122]. Liquid biopsies have emerged as a noninvasive and powerful tool for monitoring tumor progression, predicting prognosis, assessing metastasis, and evaluating drug response in cancer. Considering the remarkable stability of exosomes in various body fluids, the utilization of exosomes as biomarkers holds significant promise for OS.

## 7. Exosomes as a Vehicle for Drug Delivery in Osteosarcoma Therapy

Considering that chemotherapy remains the primary treatment approach for OS, nanomedicine research harnesses the enhanced permeability and retention (EPR) effect to facilitate drug delivery to OS, thereby decreasing chemotherapy dose, reducing associated toxicity, and targeting specific tumor sites. Apart from the traditional nanocarriers such as nanoparticles, liposomes, and polymeric micelles used for tumor targeting through the EPR effect, emerging carriers employing naturally secreted cellular vesicles exhibit enhanced biocompatibility, reduced immunogenicity, and the ability to cross various biological barriers [125,126,127,128]. Moreover, given exosome capability to transfer cargos that regulates tumorigenesis, angiogenesis, metastasis, and tumor progression, there is a growing research focus on utilizing exosomes as drug delivery vehicles. Exosomes can be engineered to carry bioactive cargos or chemotherapeutic agents (Figure 3). For example, a study by Shimbo et al. reported that upon transfection synthetic miR-143 into BMSCs, miRNA could be encapsulated into exosomes and transferred to OS cells. This exosomal cargo transport was found to inhibit cell migration in OS cells [39]. One of the challenges in utilizing miRNA therapeutics for cancer treatment is the instability of miRNAs and their short systematic half-life, attributed to their rapid renal execration. MiRNAs’ negative charge and hydrophilic characteristics present additional obstacles to crossing the cell membrane [129]. Harnessing exosomes to package miRNAs and facilitate the intercellular transport of the therapeutic cargo presents a potential for enhancing drug delivery outcomes [39]. Wei et al. developed a nanodrug, Exo-Dox, which utilizes exosomes derived from MSCs as nanocarriers for doxorubicin. Their study showed not only significantly improved cellular uptake efficiency but also better anti-tumor effects in OS cells. Notably, their study unveiled that the half-maximal inhibitory concentration (IC50) of Exo-Dox in MG63 cells was found to be lower than free Dox, suggesting the Exo-Dox holds the potential to outperform Dox in effectively treating OS cells [38]. Likewise, the utilization of exosomes derived from bone marrow MSCs to encapsulate doxorubicin exerted heightened tumor suppression and fewer side effects compared to the administration of doxorubicin as a standalone treatment in a xenograft OS model [37]. The authors hypothesized that homing capability of MSC-derived exosomes underlies the findings of both studies. Accordingly, a biodistribution analysis of labeled exosomes derived from human umbilical cord MSCs (HUC-MSCs) in OS tumor-bearing mice revealed that HUC-MSCs exosomes continuously accumulated in OS tumor sites after 24–48 h post-intravenous infusion compared to synthetic nanoparticles. Moreover, dose-dependent inhibition of OS cell proliferation was observed upon exposure to HUC-MSCs exosomes [130]. MSC-derived exosomes are recognized as multifaceted players in OS, with demonstrated roles in mediating OS progression through remodeling the TME remodeling, and functionating as drug carriers to transport therapeutic agents to the tumor site. The impact of MSC-derived exosomes on tumor behavior, whether promoting or inhibiting, is determined by the diverse cargo content present within these exosomes [37,38,63,66,78,130]. In another study, the synthetic agonist of cannabinoid receptors known as WIN was shown to induce a significant exosome secretion, and these exosomes derived from WIN-treated cells were observed to attenuate the migration of OS cells. It is worth noting that the study did not present data on exosomes from untreated cultures [131]. A recent study showed that the surface of exosomes can be engineered for targeted drug delivery. Huang et al. utilized cyclic RGD peptide (cRGD) to modify exosomes, enhancing their tumor-targeting ability. They loaded these modified exosomes with lncRNA-MEG3, a long non-coding RNA with anti-tumor properties. Their findings demonstrated that these engineered exosomes (cRGD-Exo-MEG3) exhibited improved efficiency in targeting OS cells, enhancing anti-tumor effects in vitro and in vivo [132]. Although significant progress has been made in using exosomes as drug delivery systems for cancer treatment, it is crucial to note that these studies are still in the preclinical stage. Numerous aspects require attention and continued investigations, including exosome purification, large-scale good manufacturing practice (GMP) production, and a comprehensive understanding of their mechanisms of action. One key consideration of utilizing exosomes as therapeutics is precisely controlling exosomal cargo content, as various miRNAs, proteins, DNAs, or RNAs might elicit various biological responses. Another factor is the examination of the biodistribution of exosomes to specific organs or tissues. Although early investigations suggest a favorable safety profile, evaluating exosome interaction with immune systems, potential off-target effects, and systematic responses is pivotal. Furthermore, the long-term safety of exosomes as therapeutics needs to be rigorously evaluated to ensure their efficacy and safety profiles throughout the course of cancer treatment. Nevertheless, using exosomes to deliver protein, nucleic acids, or therapeutics remains promising for advancing drug development in OS. As the field advances, thorough investigations into these aspects will not only gain deeper insights into the therapeutic potential of exosomes, but also guide us to establish robust and effective clinical applications.

## 8. Conclusions and Future Perspective

Significant progress has been achieved in understanding the pivotal role of exosomes in cancer. The information gained from the literature suggests exosomes derived from cells could mediate cell-to-cell communication, and exosomal cargos trafficking plays a role in TME, angiogenesis, metastasis, immune escape, and drug resistance. The multifaceted OS microenvironment significantly impacts disease advancement and treatment response, where exosomes from diverse cell sources like tumor cells, CAFs, MSCs, endothelial cells, and immune cells contribute by releasing a diverse array of cargos. These cargos, ranging from nucleic acids to proteins, actively modulate the progression of OS. Understanding the specific cargos carried by exosomes becomes imperative in elucidating their contribution to OS. Expanding the scope of cargo identification through next-generation sequencing not only in tumor cells but also in biological fluids holds potential for biomarker discovery in diagnosis and prognosis. In this review, we discussed the knowledge of osteosarcoma and exosome biogenesis, the regulatory mechanisms governing exosomal cargos in OS progression, their impact on immune responses and drug resistance, the promising potential of exosomes as diagnostic and prognostic biomarker tools, and the therapeutic applications of exosomes. The identification of specific exosomal cargos may serve as indicators of disease status and response to treatment. Presently, 133 clinical trials exploring the application of exosomes in cancer are underway, with two trials specifically investigating exosomes in OS. One trial involves using microfluidic chips to identify exosomes as diagnostic biomarkers for lung metastasis in OS (NCT05101655), while the other examines exosomal RNA profiles in lung metastases of primary high-grade OS (NCT03108677) [133,134].

In summary, this review provides an overview of the current state of knowledge regarding exosomes and their role in OS, highlighting the potential of inhibiting exosomes and their cargos and engineering exosomes for therapeutic drug delivery vehicles as a promising novel approach for treating OS. However, to fully harness the potential of exosomes, research efforts are required to characterize their cargos and elucidate their mechanisms of action comprehensively. This collective understanding offers a pathway to innovative diagnostic approaches and transformative therapeutic strategies in the ongoing battle against OS.

## Figures and Tables

**Table 1:** Summary of exosomal cargos and biological function in osteosarcoma.

Cargo Type	Parent Cell	Target Cells	Exosomal Cargo	Biological Function	Reference
protein	OS cells	HUVEC	VEGF-A, IL-6, IL-8	Promote angiogenesis	[33]
	OS cells	Macrophages	IL-10, TGFβ2, and CCL2	Facilitate immunosuppressive TME	[34]
	OS cells	HUVEC	VEGF, PDGF-AA, CD26, CD105, endostatin, ET-1, PAI-1, THBS1, TIMP-1, PEDF, uPA, ANG-2, TF3, PTX3, and HB-EGF	Promote angiogenesis	[40]
	OS cells	MSC	TGF-β	Induce secretion of proinflammatory cytokine IL-6	[41]
	OS cells	CAF	COL6A1	Promote OS migration, invasion, and lung metastasis	[42]
	OS cells	Macrophages	Rab22a-NeoF1	Promote lung metastasis	[43]
	BMSC	OS cells	LCP1	Promote OS proliferation, migration, invasion, and metastasis	[44]
	Adipose MSC	OS cells	COLGALT2	Promote OS proliferation, migration, and invasion	[45]
	OS cells	OS cells	Hic-5	Promote OS proliferation and inhibit apoptosis	[46]
	BMSCs	OS cells	ATG5	Promote OS proliferation, migration, invasion, and lung metastasis	[47]
	OS cells	OS cells	NPM1, CCT2, CCT4, CCT6A, CCT8, VIM, CLTC, COL6A2, HNRNPC, PKM, ACTN4, MYH10, PAICS, VCP, ANXA1, and ACLY.	Promote OS migration and invasion	[48]
	OS cells	OS cells	uPA	Promote OS migration	[49]
	OS cells	OS cells	PD-L1, N-cadherin	Promote migration, invasion, and pulmonary metastasis	[50]
	OS cells	PBMC	TGF-β	Reduce T cell proliferation and T cell activity	[51]
	OS cells	Macrophages	TIM-3	Promote OS migration, invasion, EMT, M2 macrophage polarization, and lung metastasis	[52]
	OS cells	CD3 + T cells	PD-L1	Promote tumor growth	[53]
	OS cells	OS cells	MDR-1 P-gp	Induce a doxorubicin-resistant phenotype	[54]
	OS cells	OS cells	CTCF	Enhance CDDP resistance of OS cells	[55]
miRNA	OS cells	OS cells	miR-21-5p and miR-148a-3p	Promote angiogenesis	[33]
	OS cells	HUVEC	miR-10b-5p, miR-106a-5p, miR-125b-5p, miR-143-3p, miR-145-5p, miR-146a-5p, miR-150-5p, miR-21-5p, miR-26a-5p, miR-27a-3p, miR-382-5p, miR-92a-3p, and miR-93-5p	Promote angiogenesis	[40]
	OS cells	HUVEC	miR-25-3p	Promoted invasion and angiogenesis	[56]
	OS cells	HUVEC	miR-199-5p	Inhibit HUVEC proliferation, migration, and neovascularization	[57]
	OS cells	NA	miR-21-5p, miR-143-3p, miR-148a-3p, and 181a-5p	Regulate cell adhesion and apoptosis	[58]
	OS cells	OS cells	miR-1307	Promoted OS proliferation, migration, and invasion	[59]
	OS cells	OS cells	miR-195-3p	Promote OS proliferation and invasion	[60]
	OS cells	Osteoblasts	miR-675	Promote OS migration and invasion	[61]
	CAF	OS cells	miR-1228	Promote OS migration and invasion	[62]
	BMSC	OS cells	miR-208a	Promote OS viability, clonogenicity, and migration	[63]
	BMSC	OS cells	miR-769-5p	Promote OS proliferation and metastasis	[64]
	AD-MSC	OS cells	miR-101	Inhibit OS migration and invasion	[65]
	BMSC	OS cells	miR-206	Inhibit OS proliferation, migration, and invasion; induce apoptosis; suppresses tumor growth and metastasis	[66]
	BMSC	OS cells	miR-1913	Inhibit OS proliferation, migration, and invasion	[67]
	BMSC	OS cells	miR-150	Inhibit OS cell proliferation, migration, and invasion; induce apoptosis and suppress tumor growth	[68]
	Serum	OS cells	miR-15a	Inhibit OS cell viability; promotes apoptosis and cell cycle arrests	[69]
	OS cells	OS cells	miR-144-3p	inhibits OS proliferation, invasion and migration	[70]
	OS cells	Macrophages	miR-221-3p	Enhance M2 polarization of TAMs; promote OS cell viability, migration, invasion; reduce apoptosis	[71]
lncRNA	OS cells	human microvascular endothelial cell line	lncRNA EWSAT-1	Promote angiogenesis	[72]
	OS cells	HUVEC	lncRNA OIP5-AS1	Promote angiogenesis	[73]
	BMSC	OS cells	lncRNA PVT1	Promote tumor growth and metastasis	[74]
	OS cells	OS cells	lncRNA LINC00852	Promote OS proliferation, migration, invasion, and metastasis	[75]
	Macrophages	OS cells	lncRNA LIFR-AS1	Promote OS proliferation, migration, invasion, and metastasis	[76]
	Plasma	OS cells	lncRNA CASC15	Promote tumor growth and metastasis	[77]
	BMSCs	OS cells	lncRNA MALAT1	Promote OS proliferation, migration, invasion, and tumor growth	[78]
	BMSCs	OS cells	lncRNA XIST	Promote OS proliferation, migration, and invasion	[79]
	OS cells	Macrophages	lncRNA ELFN1-AS1	Promote proliferation, migration, and invasion; promote macrophage M2 polarization and tumor growth	[80]
	OS cells	OS cells	lncRNA ANCR	Induce DOX chemoresistance	[81]
circRNA	OS cells	OS cells	circ_0000190	Inhibit OS proliferation, migration, and invasion	[82]
	OS cells	OS cells	circ_103801	Enhance chemoresistance to CDDP	[83]

**Table 2:** Exosomes as biomarker potential for OS.

Exosome Source	Exosomal Cargo	Biomarker	Number of Patients	Reference
Serum	miR-25-3p	Diagnosis and prognosis	45	[56]
Plasma	miR-92a-3p, miR-130a-3p, miR-195-3p, miR-335-5p, let-7i-3p	Diagnosis	25	[60]
Serum	miR-675	Diagnosis	8	[61]
Plasma	miR-101	Diagnosis	41	[65]
Tissue	miR-101	Diagnosis	129	[65]
Serum	lncRNA CASC15	Diagnosis	5	[77]
Tissue	lncRNA CASC15	Diagnosis	30	[77]
Plasma	circ_0000190	Diagnosis	60	[82]
Serum	TGFβ	Prognosis	10	[41]
Plasma	PD-L1, N-cadherin	Prognosis	70	[50]
Serum	circ_103801	Prognosis	43	[83]
Serum	Annexin2, CDC5L, Smad2, P27, CIP4, MTAP, PEDF, WWOX	Prognosis	93	[117]
Serum	miR-135b, miR-148a, miR-27a, miR-9, miR-124, miR-133a, miR-199a-3p, miR-385	Prognosis	93	[117]
Serum	circ_0056285	Diagnosis	35	[119]
Plasma	SENP1	Prognosis	146	[120]
Plasma	IGLV2-23, IGLV4-3, IGLV1-51, IGKV3-15, IGHV4-4, IGLV4-60, HBA1	Prognosis	40	[121]
Serum	HSATI, HSATII, LINE1-P1, Charlie 3	Diagnosis	12	[122]

## Data Availability

Not applicable.
